# How much do we care about teacher job insecurity during the pandemic? A bibliometric review

**DOI:** 10.3389/fpubh.2023.1098013

**Published:** 2023-02-09

**Authors:** Valentina Gómez-Domínguez, Diego Navarro-Mateu, Teresa Gómez-Domínguez, María del Carmen Giménez-Espert

**Affiliations:** ^1^Faculty of Education Sciences, International University of Valencia, Valencia, Spain; ^2^Department of Specific Educational Needs and Attention to Diversity, Faculty of Education Sciences, Catholic University of Valencia, Valencia, Spain; ^3^Department of Nursing, Faculty of Nursing and Chiropody, University of Valencia, Valencia, Spain

**Keywords:** job insecurity, teachers, bibliometrics, thematic analysis, COVID-19

## Abstract

In this study, a descriptive bibliometric analysis of the scientific production in the Web of Science on job insecurity perceived by teachers in pandemic situations was carried out. The result shows the growing interest in the topic with an upward trend with an annual growth of 41.52%. Forty-seven papers from 41 journals with 2,182 cited references were considered, with 149 researchers from 30 countries publishing at least one article. The country with the most publications was the United States, followed by *Germany* and *Spain*. The *United States* was the country with the most collaborations. A total of 95 institutions published papers, and the universities with the most registrations were *Miami University* and the *University of the Basque Country*, although *York University* and the *University of the Basque Country* had a higher overall citation coefficient (102 and 40, respectively). Of the 41 journals that have published on the topic, *Frontiers in Education* and the *British Journal of Educational Psychology* stood out in terms of their article numbers. However, this last one was superior in terms of the overall number of citations per year, followed by *Frontiers of Psychology*.

## 1. Introduction

The COVID-19 pandemic has had a number of consequences worldwide. One of them is the economic crisis resulting from the paralysis of the economy that occurred during the lockdown, mainly due to the closure of businesses, the stoppage of all non-essential activities and the reduction in mobility (1–3). The destruction of companies' productive capacity led to their closure in some cases, and to reductions in their workers' working hours and salaries in others (4). The pandemic has therefore had an unprecedented economic impact and has put many jobs at risk (5–7). In specific terms, the European Central Bank (ECB) shows an increase in the unemployment rate for the EU as a whole to 7.6% (2020 and 2021), reflecting a sharp deterioration in productivity, a decline in labor force growth and a clear stagnation in job creation (8). In Spain, the unemployment rate at the end of 2020 was 16.13 percent (9) and it was 13.3 percent in 2021, with a scenario of uncertainty about the duration of the crisis that is not improving, and with an inequality in terms of the widening of the wage gap for women (2 points above) and young people under 25 years of age (17.7 points above, amounting to 31% unemployment) (9, 10). This will obviously have an impact on all labor profiles, with the greatest impact on the most vulnerable groups (11). Given these circumstances, it seems logical to assume that the perception of job insecurity is becoming a topic of growing interest.

Job insecurity can be defined as the individual worker's perception of not being able to keep his or her job, or the loss of an important characteristic of the job in the face of a threatening situation (12). This insecurity is caused not only by the loss of the job itself, but also by the possibility of the event occurring, which is an even greater stressor than the loss itself (13–15). However, there are several variables that modulate reactions to job insecurity, such as economic vulnerability, psychological vulnerability and the characteristics presented by the threat (16). Reports such as the one by the Organization for Economic Cooperation and Development (OECD) (17) highlight the implications that this insecurity can cause in individuals. Fear of job loss affects the worker's psychological wellbeing and health (18). It negatively influences work engagement (19), performance (20) and creativity (21). All of this ultimately affects the productivity of companies (16) and society as a whole in the form of higher levels of general dissatisfaction, as well as increased health and social costs (22).

Although job insecurity has always been an important psychosocial risk factor in developed societies, technological, economic and political changes have increased its perception in recent times (16, 23, 24). The teaching profession has several specific characteristics such as, the prevailing diversity in the classroom, constant changes to the curriculum, and the lack of social recognition for their work, which all lead to a vulnerability in the prevalence of job insecurity and the consequences of itself (23, 25–28). However, after COVID-19, this situation and specially for teachers has been aggravated and become particularly acute (27, 28) due to the constant demands for adaptive changes and requirements which they struggle to meet (25). Among other changes suffered as a result of this situation are, the transformation of the environmental working conditions and the required working resources (29, 30), as well as the organizational characteristics of work, in both quantitative terms (the amount of work) and qualitative (knowledge and skills required) demands seen in recent years (26, 31). Regarding the latter, fear of not adapting to new technologies (32, 33) the lack of preparation and resources (20, 32, 34, 35), the lack of information and sometimes insufficient measures adopted by educational institutions, autonomous communities and central government (36) causes problems in occupational health and increases the perception of insecurity (37, 38).

It should be noted that not all levels of education are equally exposed to psychosocial risks and more specifically to job insecurity. In the case of university teaching, student autonomy is greater, and the skills required of teachers are not so directly affected, since information and communication technologies were already present before the pandemic (39, 40). However, in other stages of education, the need to adapt to new technologies and the scarcity of resources available has been a greater challenge (41–43). Likewise, student-teacher interaction is not as direct as in formal education, and the involvement and dependence on the family's participation in the teaching-learning process is also less (44). In this regard, educational attention to students with special needs and in vulnerable situations has been a major challenge in formal education (45, 46).

Given the prevalence of job insecurity as a consequence of the health emergency and in the non-university teaching profession in particular, it is important to carry out this bibliometric analysis. Ascertaining the state of the question from the articles published in the Web of Science will provide an overall perspective of the existing scientific impact, and will enable measures to improve the occupational health of teachers to be adopted. This can all contribute to mitigating the effects of the pandemic and/or administering resources and policies that reduce the negative effects on their performance in the workplace.

## 2. Materials and methods

### 2.1. Data collection

This is a bibliometric study that seeks to analyze the scientific literature in a specific field of research (47). It focuses on articles published on job insecurity in the teaching field during the COVID-19 pandemic. The research was conducted on 23 June 2022 in the Web of Science Core Collection database using the SCI-EXPANDED and SSCI indexes.

An advanced search by subject was carried out by referring to the title, abstract and keywords of the articles. The search string used in the subject field was in a first identification:

*TS* = (((“^*^employ^*^^*^certain^*^” or “^*^employ^*^^*^securit^*^”* or “career* ^*^certain^*^”* or “career* ^*^securit^*^”* or “careers* ^*^certain^*^” or “job^*^^*^certain^*^” or “job^*^^*^securit^*^”* or “labor* ^*^certain^*^”* or “labor* ^*^securit^*^”* or “labour* ^*^certain^*^”* or “labour* ^*^securit^*^”* or “métier* ^*^certain^*^”* or “métier* ^*^securit^*^” or “occupation^*^^*^certain^*^” or “occupation^*^^*^securit^*^” or “profession^*^^*^certain^*^” or “profession^*^^*^securit^*^”* or “work* ^*^certain^*^”* or “work* ^*^securit^*^”) or (^*^employ^*^ or job^*^ or labor^*^ or labour^*^ or métier^*^)) and (^*^certain^*^ *or* ^*^securit^*^) and (pandemic or covid 19 or covid-19 or covid-19 or coronavirus or “health crisis” or “sanitary crisis” or “healthcare crisis” or “health emergency” or “SARS-CoV-2”))

The search was subsequently narrowed down to teachers, and 353 articles were obtained:

(…and (teach^*^ *or school) and (pandemic or covid 19 or covid-19 or covid-19 or coronavirus or “health crisis” or “sanitary crisis” or “healthcare crisis” or “health emergency” or “SARS-CoV-2”))*

This review and selection of articles was carried out using the PRISMA (preferred reporting items for systematic reviews and meta-analyses) approach (48, 49) which is widely used in literature reviews and in various fields (50–52).

The number of records eliminated before selection was due to duplicity (*n* = 8) and based on the automation tools themselves, such as open access and/or different databases (*n* = 86), leaving a total of 259 articles to which the different inclusion and exclusion criteria were applied. The inclusion criteria were as follows: (1) literature reviews and empirical studies, (2) scientific journal articles, (3) published in any language in the last 5 years, (4) in the main collection of Web of Science, (5) addressing the job insecurity experienced by teachers and professors as a result of or during the health crisis produced by COVID-19 in their work. This resulted in 162 articles being selected.

**Figure 1:**
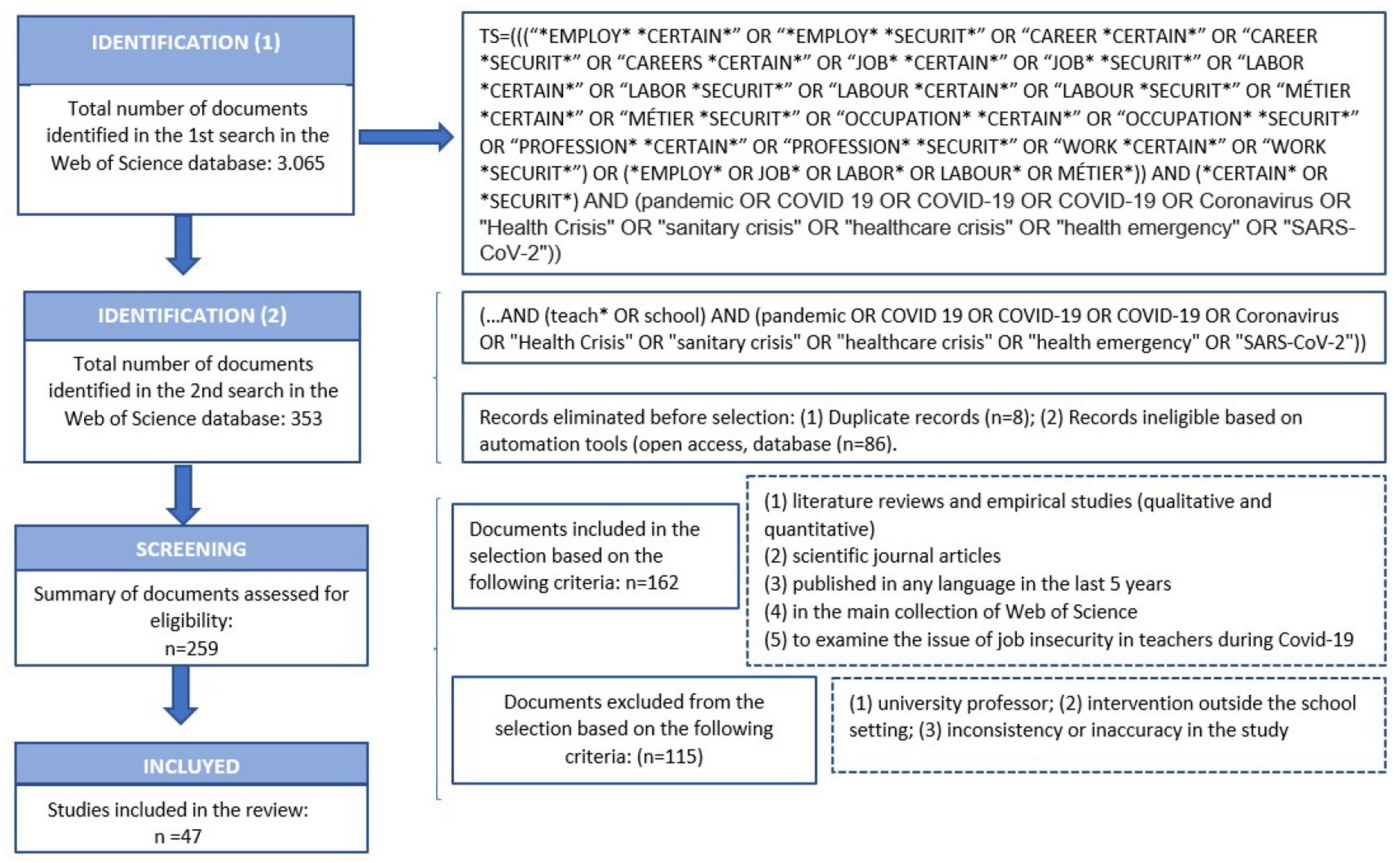
PRISMA flowchart showing the steps in source identification and selection. Adapted from Thananusak (53) and Page et al. (49).

After reviewing the content of these articles, the following exclusion criteria were subsequently applied: (1) university lecturer; (2) intervention outside the school setting; (3) inconsistency or imprecision in the study. This led to the exclusion of 115 articles and therefore to the selection of a total of 47 articles (Figure 1).

### 2.2. Bibliometric analysis

The statistical programmes used and the analyses performed with each program are detailed below:

The HistCite software package (version 2010.12.6; HistCite Software LLC, New York, NY, USA) (54) was used to calculate basic bibliometric indices, consisting of the number of articles per year, per author, per country, per institution and per journal. The information is displayed in a clear and detailed way, with quality indicators such as the total global score (TGCS) as well as the local global score (TLCS). The former refers to the total number of citations received by the articles analyzed, and the latter represents the number of citations received in WoS only by the articles selected in the specific analysis carried out (55).

The VOSviewer software package (56) was used to analyze bibliographic and thematic linkage. It enabled us to perform a bibliographic linkage analysis and the direct identification of significant articles. It is particularly useful for displaying bibliometric networks as it produces clusters that show the similarity between two or more articles by identifying the number of references they have in common. The advantage of this program is that it is not influenced by when it is used, which means it is suitable for systematic literature reviews (57).

Bibliometric analysis was performed using an R software package (58, 59). This was used to identify co-authorships, collaborations between countries and the most common keywords, as well as thematic analysis, discovering emerging, current or out-of-use topics. It allows multiple types of graphics such as networks, three-field plots, wordclouds, tree maps, historiographs, strategic diagrams, evolution maps and world maps (60).

## 3. Results

After all the documents were reviewed, the search in the WoS database retrieved a total of 47 articles published in 41 journals, by 149 authors. The average number of citations per document is 5.851. A total of 141 keywords and 192 author's keywords were found. Finally, the number of authors per paper is around 3, with an international collaboration rate of 17.02%. This information can be seen in Table 1.

**Table 1:** Main information.

**Main information about data**	
Timespan	2020:2022
Sources (Journals, Books, etc)	41
Documents	47
Annual growth rate%	41.42
Average age of document	0.864
Average citations per doc	5.851
References	2,182
**Document contents**	
Keywords plus (ID)	141
Author's keywords (DE)	192
**Authors**	
Authors	149
Authors of single-authored docs	5
**Authors collaboration**	
Single-authored docs	5
Co-authors per doc	3.3
International co-authorships%	17.02
**Document types**	
Article	44
Article; early access	3

### 3.1. Basic indicators

This first section of the results presents the basic indicators, giving details of the papers and citations per year, the number of papers and citations per author, per institution and per country. Likewise, the journals that published at least one article, the number of publications, citations and the impact factor are listed. Finally, the authors' keywords are presented according to the year of publication.

#### 3.1.1. Years

The number of published articles amounts to 47, published in 2020, 2021 and 2022. The publications per year range from six to 26, with a mean of 14.67 and a standard deviation of 8.38 (*n* = 47; range = 6–26; mean = 14.67; SD = 8.38). The first article was published in 2020, with six publications (*n* = 6). In just 1 year, there was a significant increase in the number of publications (*n* = 26) on this subject, as can be seen in the graph below. Furthermore, despite a decline in the number of publications in 2022, the trend is upwards, with an annual growth of 41.52%.

#### 3.1.2. Authors

A total of 149 researchers have published at least one article on the topic of teachers' job insecurity during the pandemic. The number of publications ranged from one to two, with a mean of 1.04 and a standard deviation of 0.20 (range = 1–2; Mean = 1.04; SD = 0.20).

**Figure 2:**
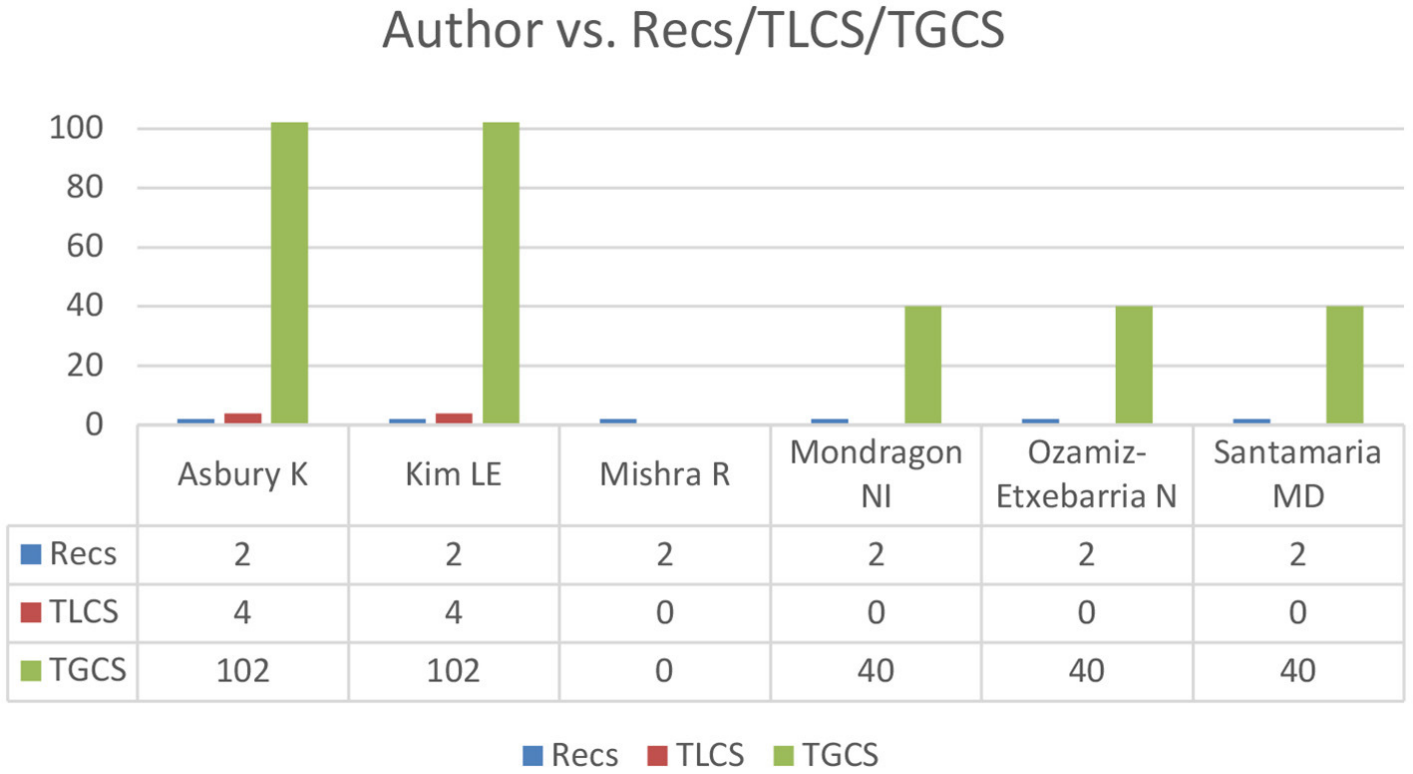
Authors with the most publications (≥2 Recs). Recs, number of articles; LCS, Local Citation Score; GCS, Global Citation Score.

The researchers with the most publications on this subject were Asbury K, Kim LE, Mishra R, Mondragon NI, Ozamiz-Etxebarria N and Santamaria MD, with two papers each. Likewise, Asbury K and Kim LE had the most overall citations with 102, followed by Mondragon NI, Ozamiz-Etxebarria N and Santamaria MD with 40. The results are shown in Figure 2, which presents the authors with the most publications and establishes two papers as the cut-off point (≥2). A comparison is also shown for the Recs, the LCS-Local Citation Score and the GCS-Global Citation Score.

These authors work in different research fields. The most common is Public Environmental Occupational Health, with eight authors (*n* = 8) followed by Education Educational Research with seven authors (*n* = 7). This is followed by Environmental Sciences with five authors (*n* = 5), Economics and Educational Psychology with four (*n* = 4) and Multidisciplinary Psychology with three (*n* = 3). Finally, Green Sustainable Science Technology, Internal General Medicine, Environmental Studies and Multidisciplinary Sciences have two authors (*n* = 2).

#### 3.1.3. Institutions

The number of institutions with publications is 95 (*n* = 95). The number of publications ranges from one to 2, with a mean of 1.03 and a standard deviation of 0.17 (range = 1–2; Mean = 1.03; SD = 0.17). Three of them have two articles and the rest have one.

**Figure 3:**
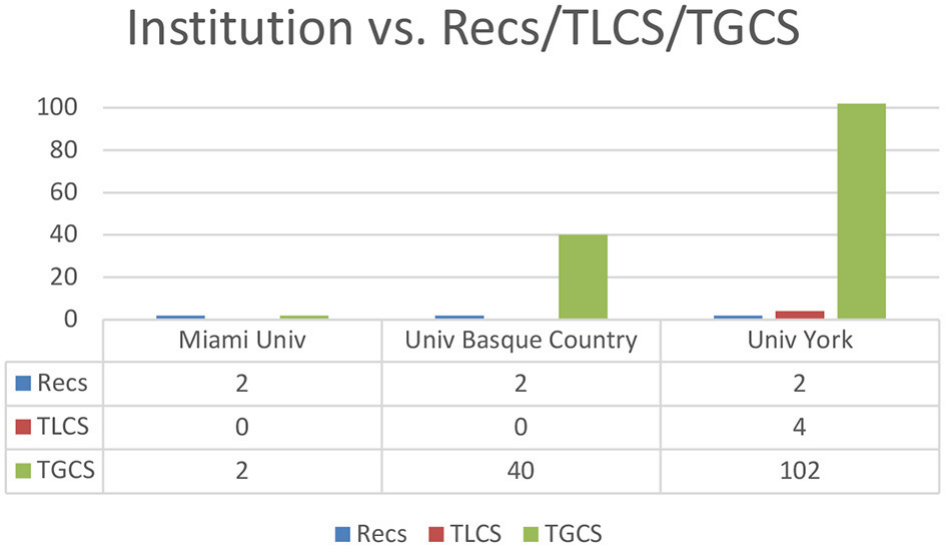
Number of publications by institution (≥2 Recs). Recs, number of articles; TLCS, Local Citation Score; TGCS, Global Citation Score.

As can be seen in Figure 3, and establishing two publications as the cut-off point (≥2), Miami University, the University of the Basque Country and the University of York are the universities with the most published papers with two papers each (*n* = 2). However, there are a total of 429 global citations, ranging from 0 to 102, with a mean of 4.52 and a standard deviation of 12.45 (range = 0–102; mean = 4.52; SD = 12.45), with 29 citations as the cut-off point (≥ 29) and the University of York has the most global citations, with a total of 102, followed by the University of the Basque Country with 40 and Greylock McKinnon Associates, IZA and St Lawrence University, with 29 total global citations.

#### 3.1.4. Countries

**Figure 4:**
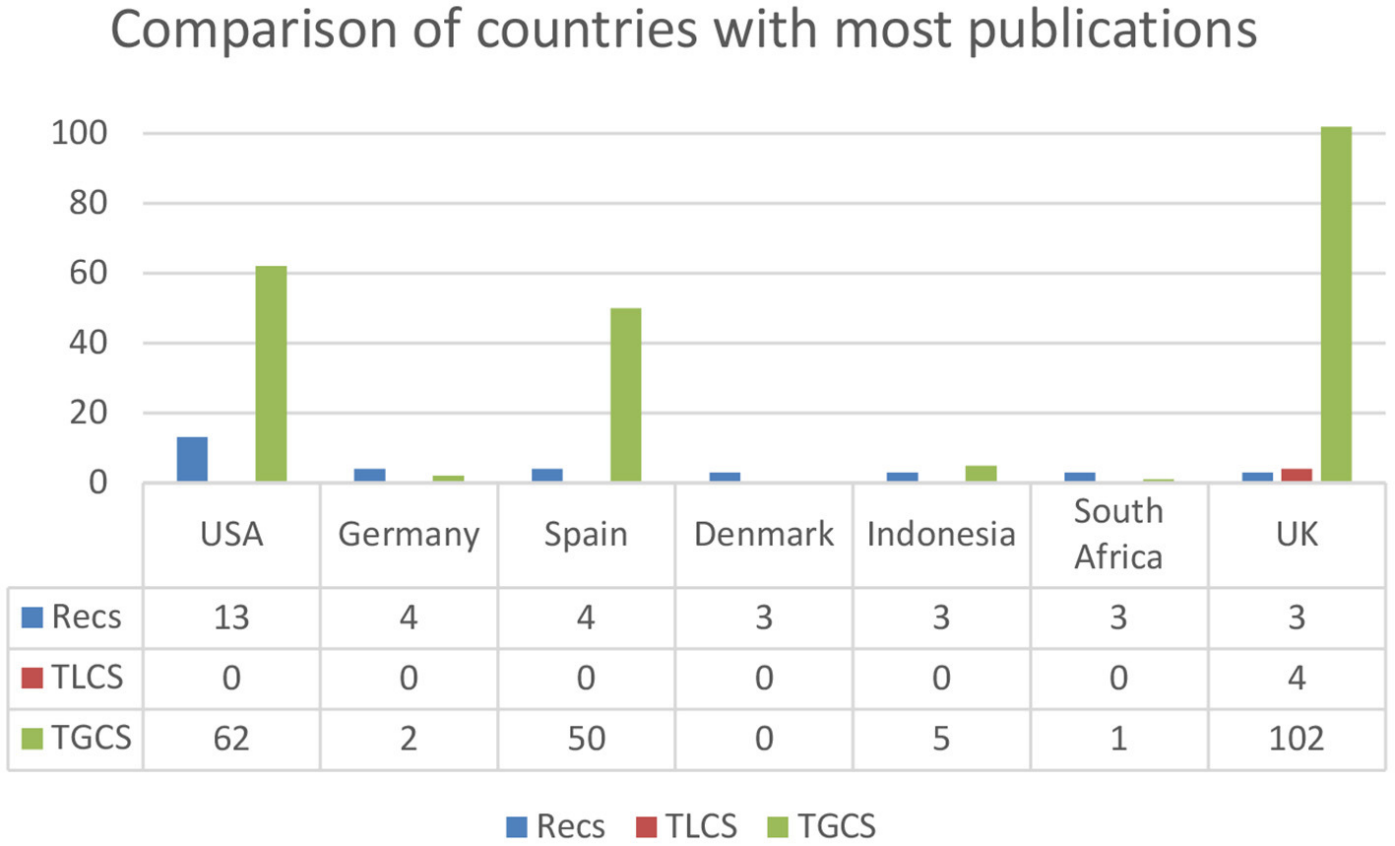
Comparison of countries with the most publications (≥3 Recs). Recs, number of articles; TLCS, Local Citation Score; TGCS, Global Citation Score.

Researchers from 30 countries have published at least one article on this Research Topic. The total number of articles is 47. The number of publications ranges from one to 13, with a mean of 2.07 and a standard deviation of 2.24 (N = 47; range = 1–13; mean = 2.07; SD = 2.24). Establishing three articles (≥3) as the cut-off point, the country with the most publications is the United States (*n* = 13), followed by Germany and Spain (*n* = 4) and finally, Denmark, Indonesia, South Africa and Great Britain (*n* = 3). This can be seen in Figure 4.

**Figure 5:**
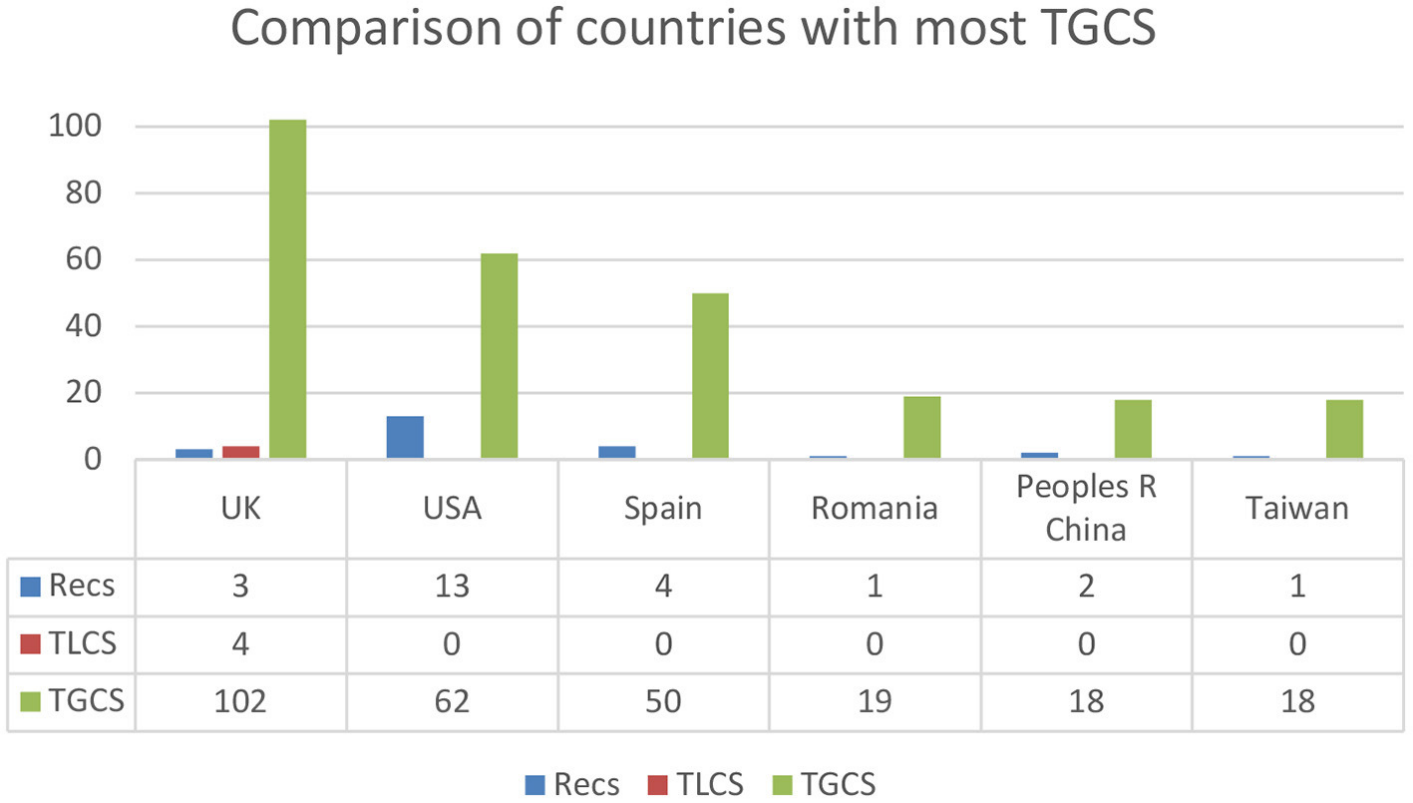
Comparison of countries with the most global citations (≥18 GCTS). Recs, number of articles; TLCS, Local Citation Score; TGCS, Global Citation Score.

The number of citations ranged from 0 to 102, with a mean of 9.97 and a standard deviation of 22.29 (range = 0–102; Mean = 9.97; SD = 22.29). The countries that have received the most citations in the WoS as a whole, with a cut-off point of more than 18 articles, are the following: Great Britain (*n* = 102), the United States (*n* = 62), Spain (*n* = 50), Romania (*n* = 19) and finally China and Taiwan (*n* = 18) (see Figure 5).

#### 3.1.5. Journals

A total of 41 journals have published at least one article on this topic. Of all these journals, four have published two articles and one journal has published more than two articles, and this indicator is the cut-off point (≥2) (Table 2).

**Table 2:** Journals by the number of publications and citations received (TLCS and TGCS) and impact factor (JCR) [76] (≥2 Recs).

**Journal**	**Recs**	**TLCS**	**TGCS**	**TGCS**	**JCR**
Frontiers in Education	3	0	5	2.50	2.32
British Journal of Educational Psychology	2	4	102	36.67	3.24
Frontiers in Psychology	2	0	38	19.00	4.23
International Journal of Environmental Research and Public Health	2	0	4	2.00	3.36
Sustainability	2	0	37	15.33	3.25

Recs, number of articles; TLCS, Local Citation Score; TGCS, Global Citation Score, TGCS/t, Global Citation Score per year;– means that these journals are in ESCI and they therefore do not yet have an impact factor.

The journals with the most articles published are Frontiers in Education (*n* = 3), followed by the *British Journal of Educational Psychology, Frontiers in Psychology, International Journal of Environmental Research and Public Health and Sustainability* (*n* = 2).

Among these five journals that have published the most articles, *Frontiers in Psychology* has the highest impact factor (JCR = 4.23), followed by *International Journal of Environmental Research and Public Health* (JCR = 3.36) and in third place by *Sustainability* (JCR = 3.25). The results can be seen in Table 2.

There is a relevant link between the articles published and the total global citations received by each journal. Table 3, shown below, shows the journals with the most total global citations, ordered from highest to lowest with a cut-off set at TGCS/t = 2. The order of relevance is changed, with the most cited being the *British Journal of Educational Psychology* (*n* = 36.67) followed by *Frontiers in Psychology* (*n* = 19), *Sustainability* (15.33), *Review of Economics of the Household* (*n* = 14.5), *Journal of Applied Psychology and Retos-Nuevas Tendencias en Educacion Fisica Deporte y Recreations* (*n* = 5).

**Table 3:** Journals by the number of total global citations per year (TGCS/t) (≥2 TGCS/t).

**Journal**	**Recs**	**TLCS**	**TGCS**	**TGCS/t**
British Journal of Educational Psychology	2	4	102	36.67
Frontiers in Psychology	2	0	38	19.00
Sustainability	2	0	37	15.33
Review of Economics of the Household	1	0	29	14.50
Journal of Applied Psychology	1	0	10	5.00
Retos-Nuevas Tendencias En Educacion Fisica Deporte Y Recreacion	1	0	10	5.00
Journal of Chemical Education	1	0	10	3.33
Accounting Research Journal	1	0	6	3.00
Frontiers in Education	3	0	5	2.50
International Journal of Engineering Pedagogy	1	0	5	2.50
Academic Pathology	1	0	4	2.00
Asia Pacific Journal of Health Management	1	0	6	2.00
International Journal of Environmental Research and Public Health	2	0	4	2.00

### 3.2. Co-citation analysis

This section presents the analysis of the co-citations. First, the co-authorship network is represented, followed by the cross-country collaboration networks, and finally the keyword networks are presented. All these results are presented in the maps below.

#### 3.2.1. Co-authorship

**Figure 6:**
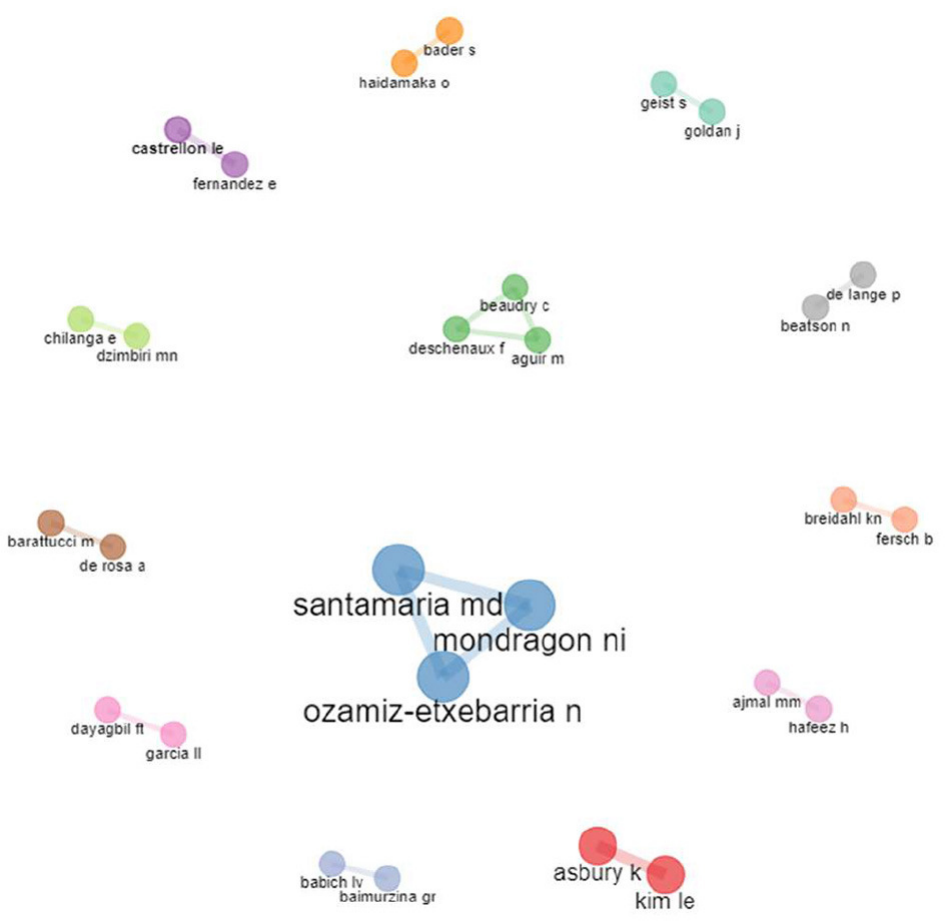
Co-authorship networks (≥1 collaboration).

Of the total of 149 authors, only collaborations between authors who have written one or more articles together are presented. The 13 co-authorship networks involving 28 researchers who have published a joint article on this topic are presented. There are two networks of three researchers, and eleven networks of two collaborators. Figure 6 shows the various collaborative networks.

#### 3.2.2. Collaborations between countries

**Figure 7:**
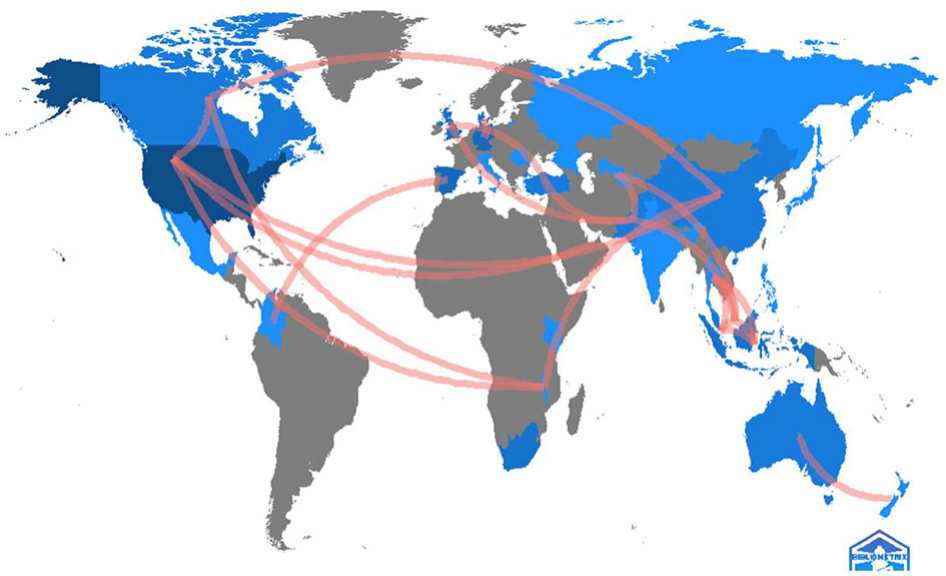
Country collaboration networks (≥1 collaboration).

As shown in Figure 7, the United States is the most collaborative country in terms of cross-country collaborations, followed by China and the United Kingdom. There are also strong collaboration networks between Spain and Colombia, and between Indonesia and Malaysia, Singapore and Thailand.

### 3.3. Thematic analysis

Finally, this third section presents the results of the thematic analysis. First, we show the bibliographic coupling analyses both by documents and by words, and second, a strategic diagram of the various themes. All these results are represented by maps.

#### 3.3.1. Bibliographic coupling for documents and keywords

**Figure 8:**
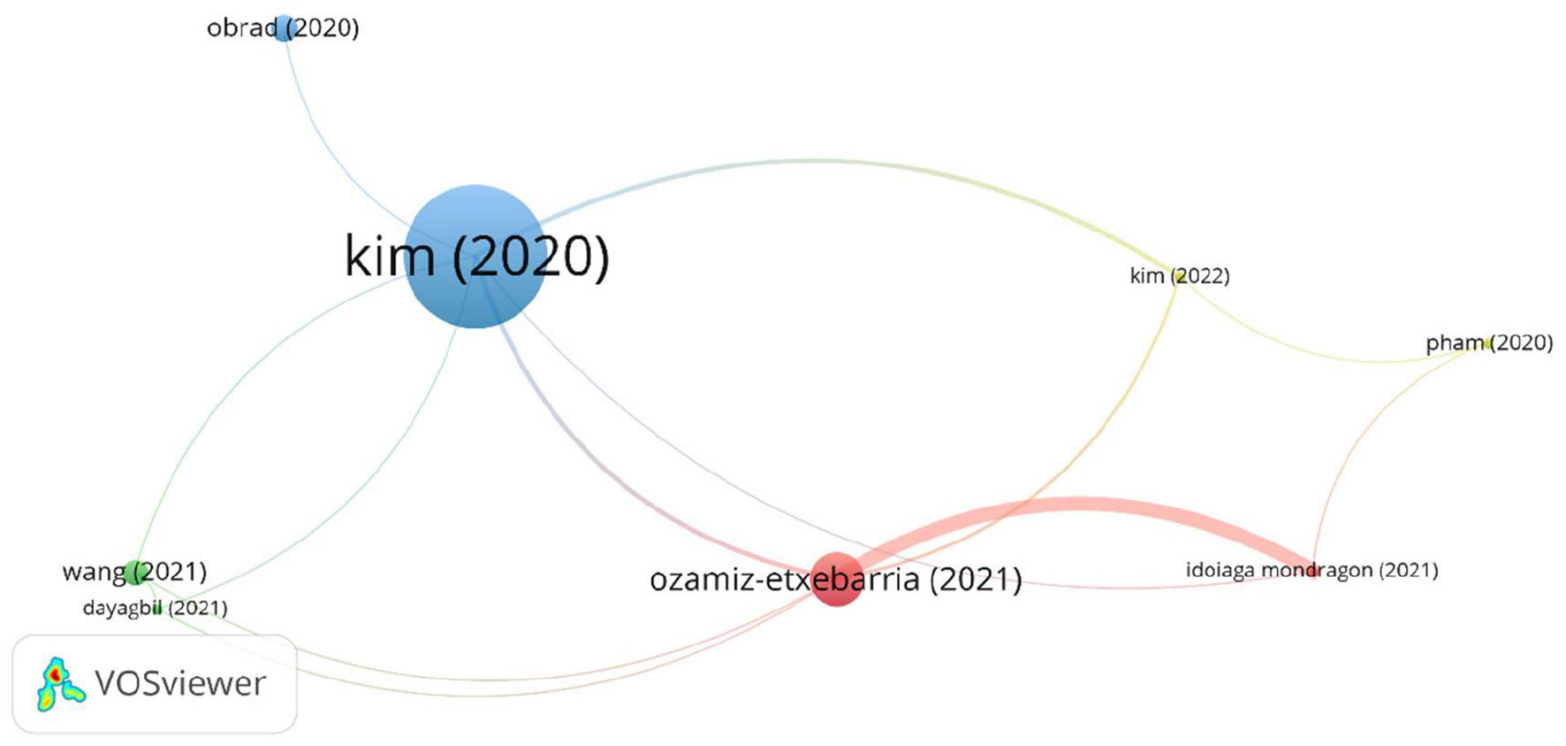
Bibliographic coupling analysis for documents (≥2 citations of publications).

A cut-off point of at least two citations per document (≥2) was established in the bibliographic coupling for documents. Subsequently, only those that were connected were selected, leaving the final analysis with eight documents, which were distributed in four different clusters (one color per cluster). The size of the letter is proportional to the number of citations and to the frequency of connections between them. These clusters are shown in Figure 8.

A thematic review of each cluster with the number of papers, citations and most prominent authors is provided below.

##### 3.3.1.1. Cluster 1-red (40 citations, two papers)

Psychological state of teachers when schools reopen. This cluster is made up of two articles (61, 62). It has received a total of 40 citations. The most cited article is Ozamiz-Etchevarria (62) with a total of 37 citations. It focuses on the level of stress, anxiety and depression experienced by teachers when they return to face-to-face work. The study shows that the period of isolation led to changes in social relations and in the working environment (62), and an increase in workloads due to having to adapt to online teaching. This increase resulted in psychosomatic problems and burnout (63). Meanwhile, the economic recession caused by the pandemic (64) has led to redundancies and instability. This situation increases the perception of job insecurity and has detrimental effects on health (62), leading to negative emotions such as a loss of confidence in their ability to teach (65). Moreover, the analysis conducted by the authors shows a relationship between high stress levels and age. Younger teachers (23–35 years) experience high levels of stress, caused by the financial uncertainty (66) which is related to job insecurity (67).

The second article is by Idoiaga Mondragón (61), with three citations. The subject is related to the psychological state of teachers with the opening of schools and the return to face-to-face teaching. According to this study the return to face-to-face teaching has led to relief of stress due to the support provided by interpersonal relationships and interaction with the teaching environment (68). These provide motivation to overcome the pandemic. Age is an important factor in measuring stress levels, and this is due to the increase in the use of new technologies (69). During the lockdown, teachers had to use them in their teaching and once they were back in the classroom, it was no longer possible to do without them. According to Idoiaga Mondragón et al. (61), older teachers have greater problems adapting to these new methodologies (70, 71). Teachers experiencing job insecurity suffer from significant psychological problems. In these cases, this psychosocial risk leads to a marked deterioration in the quality of life and consequently in the perception of safety, health and training opportunities (52, 72, 73).

##### 3.3.1.2. Cluster 2-green (21 citations, two papers)

The emergence of new technologies and the work of teachers. The theme of this cluster is the shift from traditional teaching to online teaching and its impact on teaching performance. This cluster consists of two articles. It has received a total of 21 citations. The most cited article is Wang et al. (42) with a total of 18 citations. It discusses the change in teachers' work due to the pandemic. Mainly, traditional teaching modes have had to adapt to the online context. This has some advantages that will lead to schools considering their continuity and promoting the use of platforms for online learning, as well as the development of materials and activities for this type of teaching (74).

The second article is by Dayagbil et al. (43) with three citations, which explains the adjustments that teachers had to make and their influence on teaching performance in order to be able to continue teaching in the new context. Many learners had problems in carrying out the activities because of a lack of resources and training. The authors discuss the need to take action beyond the pandemic, and to adapt a more flexible approach. This will require the adaptation of materials and new forms of assessment by teachers, as well as the provision of the resources needed for this process in terms of equipment, systems and physical structures (75).

##### 3.3.1.3. Cluster 3-blue (117 citations, two papers)

Insecurity in the face of new demands at work. This cluster consists of two papers. It has received a total of 117 citations. The most cited article is Kim and Asbury (44). These authors conduct a study of teachers' experiences of their performance during the lockdown. They discuss the need to adapt to new technologies, the uncertainty they experienced regarding the duration of this situation, and their lack of confidence in their ability to work in this new context. All of this has led them to suffer from burnout, stress and various psychological problems. The results obtained highlight the importance of interpersonal relationships with other colleagues, students and parents. Seeking emotional support from colleagues is one of the protective factors in mitigating stress. The change to remote teaching has affected these relationships, and has consequences for their self-esteem and job satisfaction (76).

The second article is by Obrad (19) and has 19 citations. This author explains the change in teachers' working conditions arising from the use of online tools and the difficulty experienced in achieving teaching objectives. The article analyzes whether teachers are able to meet the new requirements placed on them, and identifies educational constraints, stressors and factors that mediate resilience and work engagement (77).

##### 3.3.1.4. Cluster 4-yellow (10 citations, two papers)

Worsening working conditions and lack of recognition of teaching by society. Kim et al. (34) studies the demands and resources at work that teachers reported experiencing in the pandemic. This article has four citations. As a result of the closure of schools, there was a negative perception in society about the work of teachers. This perception is erroneous since despite the fact that teachers did not physically go to schools, their work did not stop—on the contrary, it increased as they had to continue their classes online. This meant an increased workload. This workload also increased when teachers returned to the classroom, as in many cases they had to manage both the students in the classroom and those who had remained at home. The study found not only an increase in workload, but also a change in the nature of the workload. The feeling of being undervalued, the burnout suffered and the impact on their mental health has led some to consider leaving the profession (78).

The article by Pham and Shi et al. (79), with six citations, performs a textual analysis of various interviews discussing the mental anguish and stress suffered from being far from their country of origin, their families, isolated from school, from the facilities as well as other factors affecting job security such as possible racism toward Asians, and a turbulent labor market.

**Figure 9:**
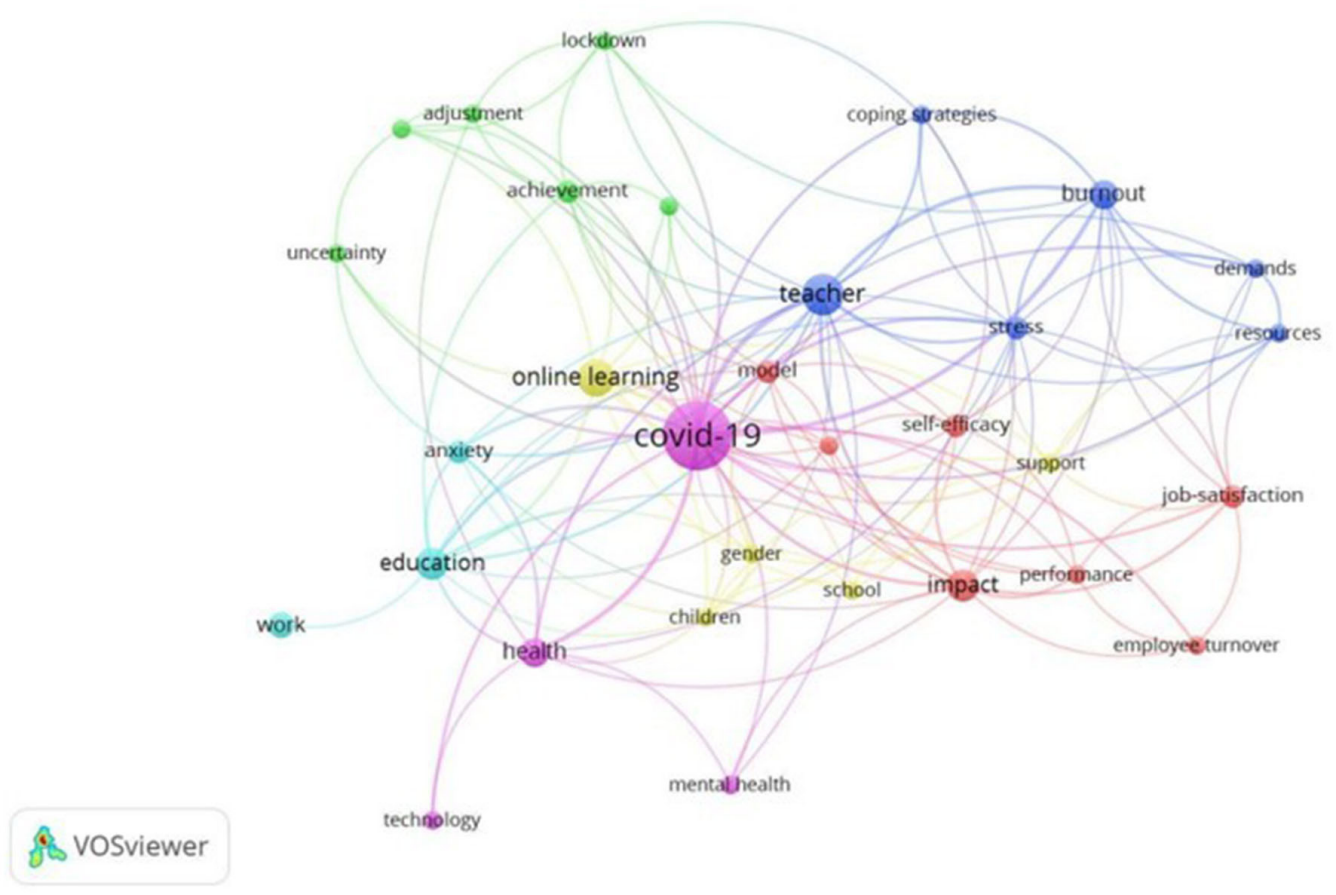
Bibliographic coupling analysis for co-word networks (≥2 co-word networks).

A bibliographic coupling for co-word networks was then performed, and a group of seven clusters of different colors is shown in Figure 9. The size of the letter is proportional to the frequency of occurrence of the keyword and to the number of connections between them in both cases.

The most common keywords used in the publications under study total 315. If the cut-off point is set at a frequency equal to or greater than two (≥2), there are 47. After screening for synonyms and groupings, we found a total of 39. Finally, after screening for non-originating words, the number is 31 (*n* = 31).

Six main groups of keywords were found. The first cluster, colored red, is composed of seven words, including “impact,” “job-satisfaction,” “life-satisfaction,” “performance,” or “self-efficacy,” and refers to the impact of the pandemic on life and work and the relationship with the perception of self-efficacy. The second cluster, colored green, contains six words, including “achievement,” “adjustment,” “information technology,” and “uncertainty.” It focuses on the need for adjustment and uncertainty, and on the need for information technology to adapt work in the classroom. The third cluster, in dark blue, is composed of six words and focuses on teacher stress, resources and coping strategies, with parameters including “coping strategies,” “resources,” “burnout” and “stress.”

The fourth one, in yellow, with eight words, refers to the use of new technologies in the classroom. There are also connected concepts such as “online learning” and “support.” A fifth violet network contains four items (“health,” “mental health,” “technology,” and “COVID-19”), referring to health and focusing on mental health. Once again, technology stands out in this grouping. The last network, in light blue, is made up of three words and refers to the anxiety suffered by teachers as a result of lockdown and related to education and work (“anxiety,” “education,” and “work”).

#### 3.3.2. Strategic thematic analysis

**Figure 10:**
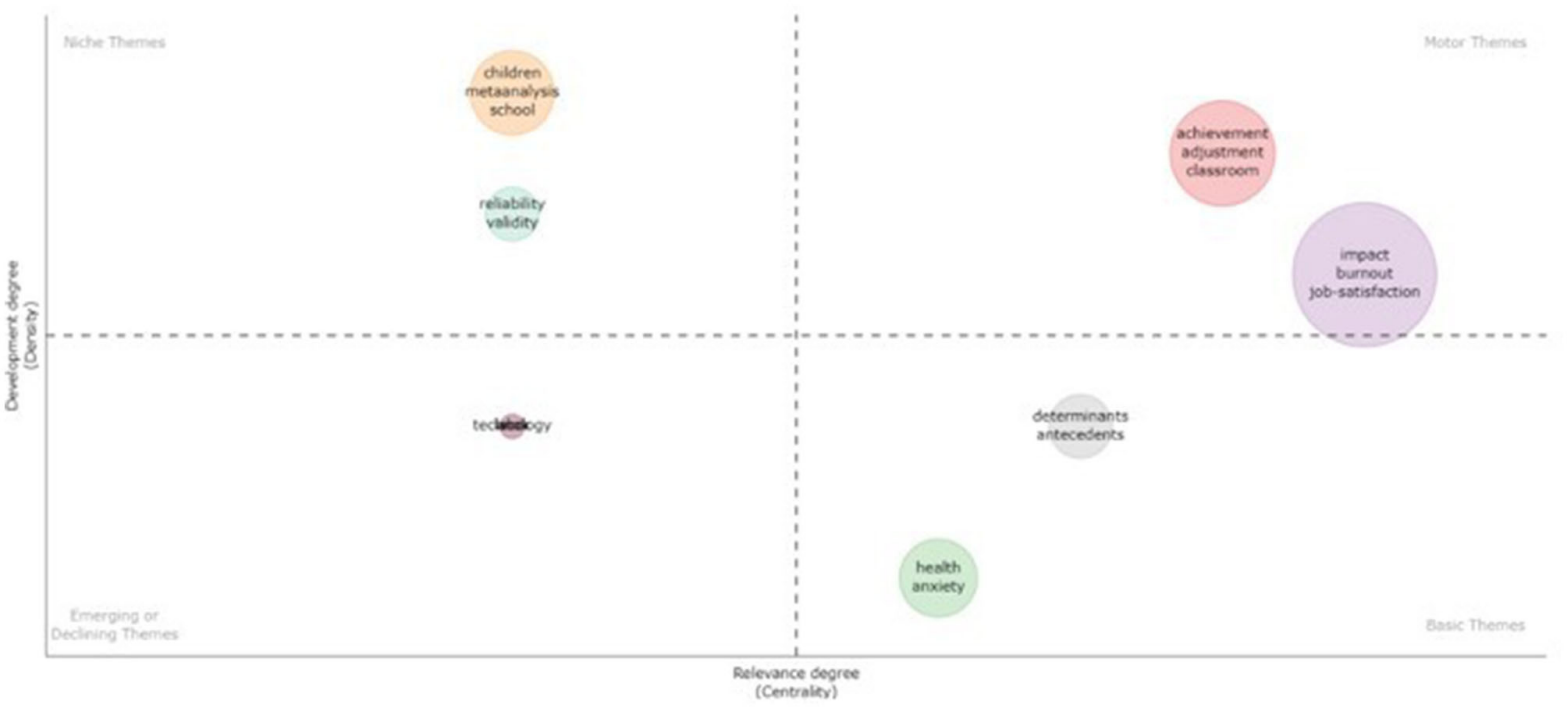
Strategic diagram of teachers' job insecurity during the pandemic.

Finally, the strategic diagram of the thematic area analyzed is presented below (Figure 10). The size of the spheres represents the number of occurrences of these keywords. The upper right quadrant shows driving themes, the upper left quadrant niche/very specialized themes, the lower right quadrant core themes and the lower left quadrant emerging or disappearing themes. The themes in the upper right quadrant are “achievement,” “adjustment,” “classroom,” “impact,” “burnout” and “job-satisfaction,” all of which are relevant and well developed for the structuring of this research field. The topics in the upper left quadrant, which are “children,” “meta-analysis,” “school,” “reliability,” and “validity” are relevant but underdeveloped, and should therefore be researched further. We can see how the latter refer to statistical aspects and this diagram therefore shows the desirability of further research of this nature.

The topics in the lower left quadrant are underdeveloped, and mainly represent emerging or disappearing topics. In this case, the term “technology” (together with “labor”) is undoubtedly an emerging theme in teaching, and its development will therefore increase considerably. The topics in the lower right quadrant are essential for this research field, and focus on the health of teachers with problems such as anxiety. As a result, the basic cross-cutting and general themes of “health,” “anxiety,” “determinants,” and “antecedents” appear in this quadrant. The thematic analysis shows that the term “technology” is increasing in importance in the field of teaching, and shows highly relevant topics that are not being sufficiently developed, and therefore need greater focus.

### 3.4. Analysis of the main results

The main results are presented below, grouped according to the methodologies used and the main thematic groups.

#### 3.4.1. Main methodological results

The majority of the research articles (55.3%) were quantitative. A total of 4.2% were triangulated, providing both quantitative and qualitative results. Meanwhile, 17% of the studies selected were qualitative in nature. Finally, 17% were literature reviews.

#### 3.4.2. Main thematic results

There are two thematic areas in relation to job insecurity. One deals with the causes, and the other with the consequences.

The causes include the following sub-themes:

- Changes in the working environment. Abandonment of a traditional model and the emergence of online learning and all that this entails. Challenges presented by online learning, lack of skills, inexperience, rapid transition without training resources, physical isolation. 36% of the publications referred to these issues (43, 80–86).
- Changes in working conditions. Increased workload with development of new materials, time devoted to training, new organization of the teaching-learning process, curricular adaptations to the new environment, among others. 30% of the publications referred to these issues (29, 30, 33, 82, 86–90).

The second thematic block focused on consequences included the following:

- Factors that modulate the consequences of insecurity. Perceived self-efficacy, social support, sense of belonging, motivation, coping strategies, resilience, interpersonal relationships, emotional intelligence and organizational support. 22% of the publications referred to these topics (20, 34, 35, 87, 90–94).
- Work-related health and attitudes: burnout, depression, stress, job dissatisfaction, fear, perceived vulnerability, uncertainty. 12% of the publications referred to these topics (19, 34, 62, 95, 96).

As has been shown, there is clearly a teaching challenge experienced when moving from traditional teaching to an online environment. The workload increases considerably, and the work demands and effort required to perform the job increases exponentially. The resources and training do not meet the requirements and as an immediate cause, teachers are subjected to stress, exhaustion and depression. In addition, fear of this new situation and working environment influences the teacher's perception of self-efficacy. Their confidence in their abilities is undermined by their lack of training and knowledge of new technologies and the tools related to them. All of this has a clear impact on fear of the new working conditions and of being unable to carry them out adequately. Some articles present job insecurity and its influence on teachers' health. Emotions such as fear, uncertainty, perceived vulnerability, make coping strategies difficult, so some publications propose techniques to improve emotional states, such as mindfulness, mindfulness and ways to enhance resilience.

Another important topic due to its relevance in education is the educational response to students with special educational needs (93, 97, 98), which is addressed in three articles. This type of student requires direct and individualized attention. Personal contact with these students is necessary, and online teaching does not enhance this aspect. Their learning and development are clearly impaired in this new working environment.

Finally, several authors stress the importance of the permanence of online methodologies in teaching (33, 42, 43). The advantages of online teaching require schools to assume that they will continue and with it, the need to promote the use of educational platforms and materials for online learning (29, 33, 34, 87, 92).

## 4. Discussion

This article analyzes the importance of job insecurity among teachers since the beginning of the pandemic. It also performs a critical assessment of the publications on this subject in order to evaluate the impact of COVID-19 on the work environment of teachers, and how it has affected their psychosocial risks and their job security in particular.

Interesting data can be extracted from the analysis carried out. Since the subject matter is related to the COVID-19 pandemic, the first article was published in 2020. There were six publications in that year, and there was a significant increase in the number of publications in a single year, to 26 in 2021. In 2022, despite the time elapsed since the start of the pandemic and a decline compared to 2021, this topic continued to be of interest, with 12 publications up to the time of the search. The most prolific institutions, with two publications each, were *Miami University*, the *University of the Basque Country* and the *University of York*. In terms of global citations, the *University of York* has the most global citations, with a total of 102, followed by the *University of the Basque Country* with 40. Although the number of publications is low, the number of citations is high, which shows the interest the articles attract. The most prolific countries are the United States with 13 publications, followed by Germany and Spain with 4. The United Kingdom was the country with the most citations, with 102 global citations, followed by the United States and Spain, with 62 and 50 respectively. The United States was the country with the most collaborations, followed by China and the United Kingdom. This rate of collaboration is positive, as this is a pandemic that affects the whole world, and sharing information can be highly beneficial (99).

A total of 149 authors have published articles related to this issue, with Asbury K, Kim LE, Mishra R, Mondragón NI, Ozamiz-Etxebarria N and Santamaria MD being the most prolific authors with two papers each. The most collaborative authors are in two groups: Santamaria, Mondragon, Ozamiz-Etxebarria, and another group formed by Beaudry, Deschenaux and Aguir. The most common fields to which the authors interested in the question belong are Public Environmental Occupational Health with eight authors, followed by Education Educational Research with seven authors. It should be noted that although the number of publications is not excessively high, the number of global citations is high, rising to 102 in the case of Asbury K and Kim LE and 40 in the case of Mondragón NI, Ozamiz-Etxebarria and Santamaria MD. This shows the growing interest in the subject. The review carried out shows that some authors focus on the causes that have led teachers to perceive job insecurity, analyzing the changes in working conditions that have appeared since the pandemic.

The results show that changing working conditions and lack of confidence in the ability to adapt to the new environment will increase the perception of job insecurity. This perception is due not only to a loss of employment, but also to the fear that it may occur (32, 61, 80). These results are consistent with the general literature on job insecurity (12–16). This psychosocial risk will cause stress in teachers and if sustained over time, lead to burnout, various psychosomatic disturbances and health problems (37). One of the recurring points addressed in the articles by the researchers (20, 29, 33–35, 42, 43, 80, 82, 83, 87, 88, 100, 101) is the perception of being unable to adapt to the use of the technologies needed to meet new labor demands. Adapting to this new context requires time and effort in training, the use of innovative resources, the creation of new materials and the provision of space, which must be combined with every day and family life (20, 29, 30, 34, 42, 87). Other articles discuss the transition from traditional teaching to online teaching, focusing on the consequences such as increased workloads (29, 30, 82, 88). Teachers have to adapt to the new online context by creating materials, interactive activities, new forms of assessment and providing adequate resources (42). This workload and its changing nature sometimes leads to a decline in perceived self-efficacy (20, 30, 44). This has an impact on job security, and leads teachers to suffer from burnout and even leave the profession (34). The closure of schools during the health emergency period led to the need to adapt to this new work context. This situation causes technostress (80) as the worker perceives that they are unable to adequately handle the demands made of them (102).

One author, Koç and Fidan (81), sees significant distinctions between teachers in private and public sector institutions. According to this author, teachers in public schools are more likely to acquire digital competences in order to adapt to the new situation. Job insecurity also moderates the relationship between technostress and teachers' willingness to use online modalities, with temporary contract workers being more likely to use new technologies (80). Other studies, such as the one by Ozamiz-Etxebarria et al. (62), show that teachers with temporary contracts of less than 3 months have the highest levels of depression and anxiety, with the percentages of pre-school, primary and secondary school teachers being higher than among university teachers (23). Meanwhile, there are articles that point out the factors that modulate the impact of job insecurity and psychosocial risks on teachers' health (44). These factors include peer emotional support and the establishment of significant support structures (61, 91, 92). These were undermined by the social isolation that occurred at the height of the pandemic. Another modulating factor is the provision of skill-building resources that enhance perceptions of self-efficacy (32, 81, 87). One such resource is the provision of support for remote learning (90). Another protective factor is fostering organizational support (30, 34). Involving teachers in school decisions is one of the measures that contributes to an increased sense of control (98) and lowers levels of insecurity and stress. All these factors contribute to alleviating emotional exhaustion and promote coping strategies (103) that help to improve stress levels and mental health.

These results imply progress in the field of non-university teachers who currently lack bibliometric studies on job insecurity in general (104) and on conditions in a health emergency such as COVID-19 in particular. However, the study is not without its limitations. The first one concerns using a single WoS database even though it is a controlled and verified quality source (105). The second is the limited number of publications that make direct reference to the job insecurity experienced by teachers as a result of the pandemic. As a proposal for the future, we suggest that a more extensive compilation covering other databases should be carried out, grouped and evaluated using a methodological quality tool such as a meta-analysis. Furthermore, it would be interesting to extend the study to other psychosocial risks, and to determine the significance and relationship between them and job insecurity. This study would make it possible to propose strategies to improve the health and working conditions of teachers, and to mitigate the effects of the psychosocial risks they experience.

## 5. Conclusion

This bibliometric analysis provides an objective measurement of the topics of most interest to researchers, collaborations between researchers, citations, the impact factor of journals, and the countries and institutions most interested in the issue. The impact of COVID-19 on the job insecurity of teachers is of interest to researchers but needs further investigation.

The results focus mainly on the causes and consequences of job insecurity, as well as on the moderating variables (social support, interpersonal relationships, organizational support, etc.) that make it possible to mitigate the effects of this psychosocial risk. As well as on the existence of job insecurity determinants such as organizational justice, organizational support or perceived self-efficacy. These findings indicate that school administrators and policymakers can intervene to address the job insecurity of their non-university teachers by fostering organizational support, involving teachers in school decisions, and helping to increase their sense of control. All these factors contribute to alleviating emotional exhaustion and promote coping strategies to improve the level of stress and mental health of non-university teachers, thereby benefiting society as a whole.

## Author contributions

Conceptualization: VG-D and DN-M. Methodology and formal analysis: MdCG-E and TG-D. Software and project administration: VG-D. Investigation: DN-M. Resources and writing—review and editing: VG-D and MdCG-E. Writing—original draft preparation and visualization: DN-M, VG-D, and TG-D. Supervision: MdCG-E. All authors have read and agreed to the published version of the manuscript.
